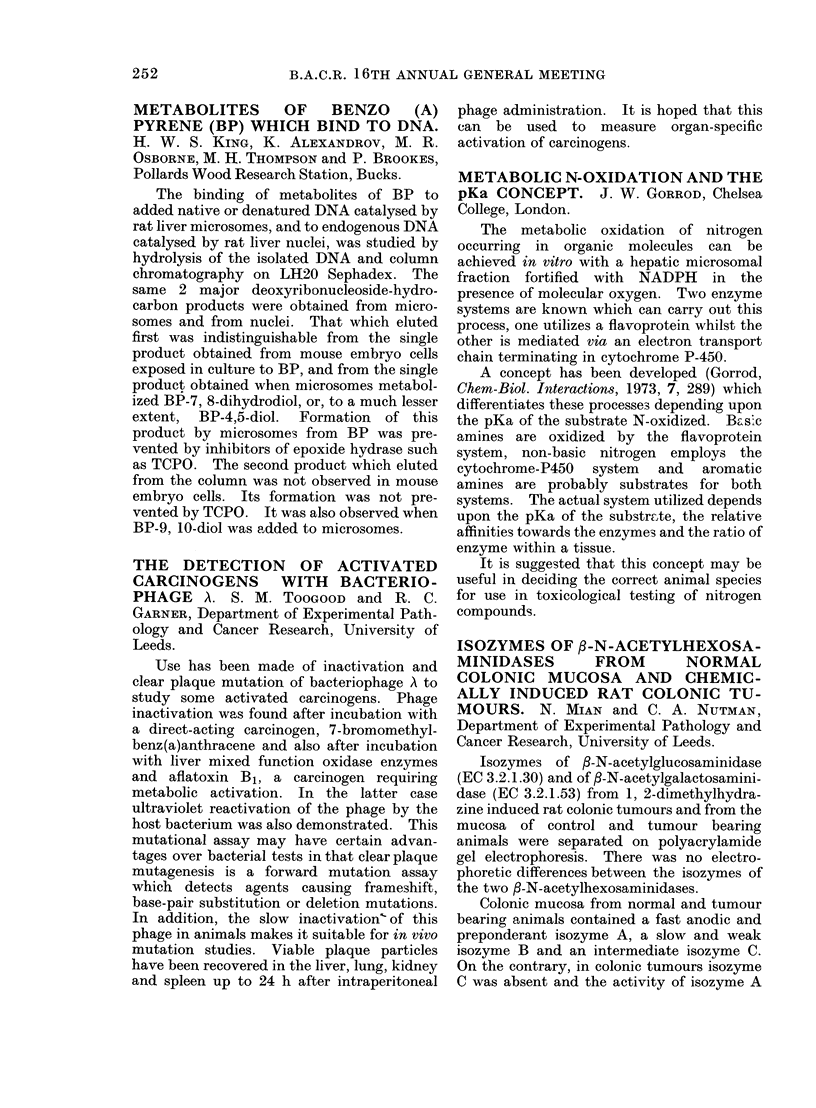# Proceedings: The detection of activated carcinogens with bacteriophage lambda.

**DOI:** 10.1038/bjc.1975.195

**Published:** 1975-08

**Authors:** S. M. Toogood, R. C. Garner


**THE DETECTION OF ACTIVATED CARCINOGENS WITH BACTERIOPHAGE $\lambda$.** S. M. Toogood and R. C. Garner, Department of Experimental Pathology and Cancer Research, University of Leeds.

Use has been made of inactivation and clear plaque mutation of bacteriophage $\lambda$ to study some activated carcinogens. Phage inactivation was found after incubation with a direct-acting carcinogen, 7-bromomethylbenz(a)anthracene and also after incubation with liver mixed function oxidase enzymes and aflatoxin $B_1$, a carcinogen requiring metabolic activation. In the latter case ultraviolet reactivation of the phage by the host bacterium was also demonstrated. This mutational assay may have certain advantages over bacterial tests in that clear plaque mutagenesis is a forward mutation assay which detects agents causing frameshift, base-pair substitution or deletion mutations. In addition, the slow inactivation of this phage in animals makes it suitable for *in vivo* mutation studies. Viable plaque particles have been recovered in the liver, lung, kidney and spleen up to 24 h after intraperitoneal phage administration. It is hoped that this can be used to measure organ-specific activation of carcinogens.